# Topical tranexamic acid powder for bleeding control in dentistry: a scoping review

**DOI:** 10.31744/einstein_journal/2026RW1859

**Published:** 2026-03-13

**Authors:** Beatriz Rezende Bergo, Letícia Mendes Nunes, Camila Vassallo de Andrade, Paula Cristina Costa, Francisca Daniele Moreira Jardilino, Amanda Leal Rocha

**Affiliations:** 1 Universidade Federal de Minas Gerais Belo Horizonte MG Brazil Universidade Federal de Minas Gerais, Belo Horizonte, MG, Brazil.

**Keywords:** Tranexamic acid, Surgery, oral, Hemostasis

## Abstract

**Objective:**

This scoping review evaluated the efficacy of topical tranexamic acid powder, prepared from crushed tablets, for bleeding prevention during minor oral surgical procedures.

**Methods:**

A comprehensive literature search was conducted in accordance with PRISMA-ScR guidelines across MEDLINE/PubMed, Elsevier, Embase, BVSalud, Web of Science, and Periódicos CAPES. Studies investigating topical tranexamic acid in powder form for minor dental procedures were included. Reviews, *in vitro* or animal studies, and investigations focusing on systemic, injectable, or mouthwash formulations of tranexamic acid were excluded. Methodological quality was assessed using the Newcastle-Ottawa Scale and Joanna Briggs Institute critical appraisal checklists.

**Results:**

Ten studies met the inclusion criteria, including clinical trials, retrospective and prospective studies, case reports, and one letter to the editor. Tranexamic acid powder was applied directly to surgical sites or prepared as a paste or saline suspension, with doses ranging from 250 to 500mg. Most participants were receiving anticoagulant therapy or had underlying bleeding disorders, highlighting the need for effective local hemostatic approaches. All included studies reported successful bleeding control with topical tranexamic acid, frequently combined with additional local hemostatic measures such as sutures or gelatin sponges.

**Conclusion:**

Topical tranexamic acid powder shows promise as an adjunctive measure for bleeding control in minor oral surgery, particularly in individuals at increased risk of hemorrhage. However, further studies are needed to clarify the isolated efficacy of tranexamic acid powder in dental bleeding management.

**Open Science Framework:** https://osf.io/n5mbp

## INTRODUCTION

Tranexamic acid (TXA) is a synthetic lysine derivative and an antifibrinolytic agent widely used to reduce bleeding.^(1,2)^ Pharmacokinetic and mechanistic studies show that TXA inhibits fibrin degradation by competitively blocking lysine-binding sites on plasminogen, thereby preventing plasmin formation.^(2)^ This action stabilizes established clots and limits fibrinolysis, making TXA a valuable agent for bleeding control in clinical practice.^(3)^ Tranexamic acid is available in multiple formulations for different routes of administration, including injectable solutions, pills, oral tablets, mouthwash preparations, and topical gels.^(2–4)^

The medical literature indicates that intravenous TXA effectively reduces bleeding during cardiovascular and orthopedic surgeries and is commonly used for hemostasis in hemorrhagic complications.^(5)^ However, systemic administration of TXA, whether oral or intravenous, may cause adverse effects related to generalized inhibition of fibrin degradation.^(2,6)^ Consequently, local application of TXA to the bleeding surface has emerged as a viable strategy to reduce bleeding while limiting systemic exposure.^(7,8)^ In cardiovascular surgery, intrapericardial topical application of TXA in powder form has demonstrated efficacy in reducing postoperative bleeding and has been shown to be comparable to injectable formulations, without an associated increase in postoperative seizure risk.^(9)^

In dentistry, a ready-to-use 5% TXA mouthwash is commercially available in several countries.^(2,4)^ This formulation effectively controls postoperative bleeding following oral surgery, particularly in patients receiving anticoagulant therapy.^(2–4)^ In settings where a commercial TXA mouthwash is unavailable, a mouth rinse can be prepared by diluting the intravenous formulation with water or by creating a suspension from TXA tablets.^(2)^ Alternatively, crushed TXA tablets mixed with saline can be applied directly to the surgical site to control intraoperative bleeding.^(4,10)^

**Figure 1:**
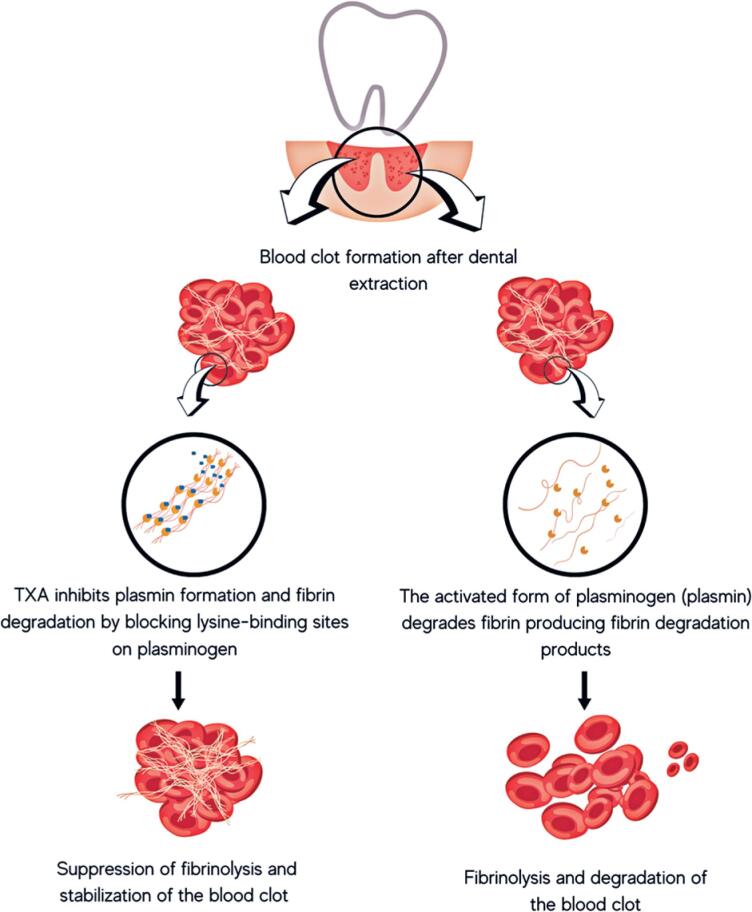
Antifibrinolytic effect of tranexamic acid (TXA) versus no tranexamic acid following dental extraction

Despite the well-established use of 5% TXA mouthwash, evidence on the local application of TXA tablets in dentistry remains limited. This review addresses this underexplored approach and contributes new insight, particularly given the increasing off-label use of TXA formulations. The aim of this study was to conduct a scoping review evaluating the efficacy of topical TXA powder in patients undergoing minor oral surgery for the prevention of bleeding and hemorrhagic complications (Figure 1).

## METHODS

### Protocol

The search was conducted in October 2024 by four independent, calibrated reviewers. Rayyan software (Rayyan Systems, Toronto, Canada) was used to screen and filter records retrieved from electronic databases.^(11)^

### Focused question

This study addressed the following focused question: What is the efficacy of topical TXA powder in controlling bleeding and preventing hemorrhagic complications following dental surgery?

### Study design and eligibility criteria

Clinical studies, case reports, and letters to the editor involving patients undergoing oral surgery and reporting the topical use of crushed TXA tablets were included.

### Exclusion criteria

Reviews, conference abstracts, editorials, *in vitro* studies, animal studies, and studies describing topical TXA use as ready-to-use mouthwash solutions, injectable formulations, or systemic administration rather than crushed tablets were excluded. Studies focusing on major surgical procedures, such as cardiovascular or orthopedic surgery, were also excluded.

### Intervention

The intervention consisted of adjunctive hemostatic measures during or after dental surgical procedures, specifically the topical application of TXA prepared from tablets. The tablets were either crushed and applied directly to the surgical site or diluted in saline, with evaluation focused on bleeding control.

### Outcome measures

The review assessed the effectiveness of powdered TXA in controlling intraoperative hemorrhage and managing postoperative bleeding, building on the established efficacy of other formulations. Application techniques were compared, including direct placement of crushed tablets, paste preparations, and aqueous dilutions. Dosage, frequency of application, and the type of oral procedure—such as biopsy, dental extraction, and periodontal surgery—were also examined to evaluate the efficacy of TXA across different clinical settings.

### Information sources

The search was conducted across several major electronic databases with comprehensive coverage of medical and scientific literature, including the National Library of Medicine MEDLINE/PubMed, Scopus, Embase, BVSalud, Web of Science, and the *Portal de Periódicos da Coordenação de Aperfeiçoamento de Pessoal de Nível Superior* (CAPES). Reference lists of included studies were also screened. Duplicate records identified across databases were removed using EndNote software. No restrictions were applied regarding language, publication date, or geographic region.

### Search strategy

A comprehensive search strategy was developed to identify studies evaluating the topical or local application of TXA at surgical sites. The strategy was adapted for each database to maximize retrieval of relevant articles. Searches were performed using title, abstract, and keyword fields in all databases (Table 1S, Supplementary Material).

### Study selection and data extraction

Two independent, blinded reviewers conducted all stages of the literature search using Rayyan software. Studies with titles and abstracts meeting the eligibility criteria were included. When titles or abstracts were unavailable or provided insufficient information, full texts were retrieved and assessed. Articles fulfilling the inclusion criteria were selected accordingly. Disagreements between reviewers were resolved through discussion with a third reviewer until consensus was achieved.

The following data were extracted from each included study: first author's surname, year of publication, country of origin, study design, sample size and study groups, type of surgical procedure, references related to TXA, method of topical TXA application, and outcomes related to bleeding control.

### Critical appraisal of studies

Quality assessment was performed independently by two reviewers, with disagreements resolved through discussion. The included studies were evaluated using validated tools to assess risk of bias. For cohort, case-control, prospective, and retrospective studies, the Newcastle-Ottawa Scale^(12)^ was applied in accordance with Cochrane Collaboration guidance for non-randomized studies. This scale evaluates three domains: participant selection, group comparability, and outcome assessment. Studies received one star for each criterion met, with higher scores indicating lower risk of bias; 7-9 stars indicated low risk, 4-6 stars moderate risk, and fewer than 4 stars high risk.

The Joanna Briggs Institute (JBI) Critical Appraisal Checklists^(13)^ were used for clinical trials and case reports. For clinical trials, the checklist assessed allocation concealment, blinding, similarity of treatment between groups, completeness of follow-up, appropriateness of statistical analysis, and clarity of outcome reporting. For case reports, the JBI checklist evaluated clarity of patient information, clinical history, diagnostic methods, treatment, outcomes, adverse events, relevance of discussion, and clinical implications. Studies meeting at least 70% of JBI criteria were classified as low risk of bias, those meeting 40%-69% as moderate risk, and those meeting less than 40% as high risk (Table 2S, Supplementary Material).

### Protocol and registration

This review was conducted and reported in accordance with the Preferred Reporting Items for Systematic Reviews and Meta-Analyses Extension for Scoping Reviews (PRISMA-ScR) guidelines. The study protocol was registered in the Open Science Framework (https://osf.io/n5mbp).

## RESULTS

### Study selection

**Figure 2:**
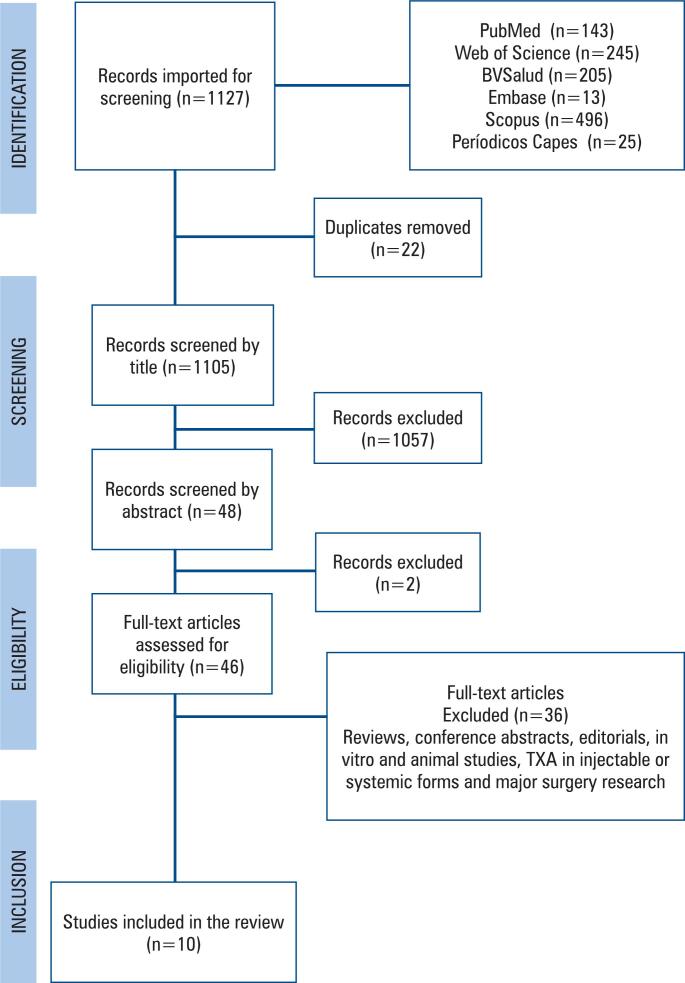
Flow diagram of the study selection process

A total of 1,127 records were identified across electronic databases: 143 from PubMed, 245 from Web of Science, 205 from BVSalud, 496 from Scopus, 25 from Periódicos CAPES, and 13 from Embase. After removal of 22 duplicates, 1,105 titles and abstracts were screened. Of these, 1,057 records provided insufficient information for eligibility assessment and were therefore excluded. Forty-eight articles met the eligibility criteria based on title and abstract and were retrieved for full-text review. Two articles were excluded at this stage for not meeting the inclusion criteria. Full-text assessment was performed for 46 articles, of which 36 were excluded for predefined reasons. Ultimately, 10 studies were included in the review.^(4,10,14–21)^ The study selection process is illustrated in figure 2.

### Analysis of study types for selected articles

Of the 10 included studies, three were retrospective studies, two were clinical trials, and two were prospective cohort studies. The remaining three articles comprised one case-control study, one case report, and one letter to the editor (Table 1). All included articles underwent critical appraisal to assess risk of bias (Table 2S, Supplementary Material).

**Table 1:** Comprehensive overview of topical tranexamic acid protocols

Author, year	Country	Study design	Sample size and study groups	Type of dental procedure	TXA administration methods	Outcomes*
Bernardoni-Socorro et al., 1998 ^(14)^	Venezuela	Clinical trial	Patients receiving anticoagulant therapy (n=15) treated with TXA (n=19 procedures) and a control group B without additional hemostatic measures (n=18 procedures)	Tartar removal, curettage, and dental extraction	A TXA tablet (250 mg) was crushed and diluted in 10 mL saline for postoperative bleeding control. TXA mouthwash was used for three minutes every six hours for seven days	TXA reduced bleeding in anticoagulated patients during dental procedures, including those with periodontal disease, without interruption of anticoagulant therapy
Blinder et al., 1999 ^(18)^	Israel	Clinical trial	Patients receiving anticoagulant therapy divided into three groups: RS and sutures (n=50); RS, sutures, and TXA mouthwash (n=50); and fibrin glue with RS and sutures (n=50)	Dental extraction	Local pressure was applied using gauze soaked in a TXA solution (500mg) diluted with saline. An RS was placed in the alveolar socket. For postoperative bleeding control, TXA mouthwash (500mg) was used for two minutes, four times daily for four days	Dental extractions were safely performed in anticoagulated patients using hemostatic measures such as RS, sutures, and TXA powder. However, severe periodontitis was associated with an increased risk of bleeding
Coetzee, 2007 ^(19)^	South Africa	Letter to the editor	Patients with hemophilia (n=30)	Dental extraction	TXA tablets (500mg) were crushed and placed on wet cotton wool positioned over the extraction site, and patient were instructed to apply pressure by biting for 30 minutes	Crushed TXA tablets could offer a cost-effective option for bleeding control in resource-limited settings, although further studies are required to confirm their effectiveness
Buhatem Medeiros et al., 2017 ^(10)^	Brazil	Case-control study	Patients receiving antiplatelet therapy (n=38) and a control group (n=35)	Dental extraction	A paste prepared from a crushed TXA tablet (250mg) mixed with saline was applied to the dental alveolus	Dual antiplatelet therapy was maintained during dental extraction. Although bleeding intensity was greater, bleeding was controllable using local measures, including crushed TXA tablets applied at the surgical site
Rocha et al., 2017 ^(15)^	Brazil	Retrospective study	Patients receiving anticoagulant therapy (n=126), antiplatelet therapy (n=53), or dual therapy (n=14)	Dental extraction	A TXA paste prepared from a crushed 250-mg tablet mixed with saline was used to soak the RS filling the alveolar socket. For postoperative bleeding control, TXA mouthwash (one tablet in 100 mL cold saline) was used four times daily for seven days	Postoperative bleeding was minimal in both groups. Local hemostatic measures, including TXA paste and RS, controlled bleeding without interruption of antithrombotic therapy
Lu et al., 2018 ^(21)^	Taiwan	Retrospective study	Patients receiving antiplatelet therapy (n=183) or VKA therapy (n=60)	Dental extraction	The extraction socket was repacked with Gelfoam impregnated with TXA powder. For postoperative bleeding control, TXA mouthwash used over several days was a convenient option	Interrupting warfarin therapy before dental extraction did not significantly reduce bleeding risk. Local hemostasis was achieved using crushed TXA tablets and other local measures
Rocha et al., 2019 ^(4)^	Brazil	Prospective cohort	Patients receiving VKA therapy (n=75) and non-anticoagulated controls (n=77)	Dental extraction	A TXA paste prepared from a crushed 250-mg tablet mixed with saline was used to soak the RS filling the alveolar socket. For postoperative bleeding control, TXA mouthwash (one tablet in 100mL cold saline) was used four times daily for seven days	Bleeding during dental extraction was similar between groups. TXA effectively controlled bleeding when used alongside additional local hemostatic measures
Rocha et al., 2020 ^(16)^	Brazil	Prospective cohort	Patients receiving DOACs (n=11) or VKA therapy (n=15) and non-anticoagulated controls (n=15)	Dental extraction	A TXA paste prepared from a crushed 250-mg tablet mixed with saline was used to soak the RS filling the alveolar socket. For postoperative bleeding control, TXA mouthwash (one tablet in 100mL cold saline) was used four times daily for seven days	Patients receiving DOACs exhibited a low risk of bleeding. TXA paste, used as part of a comprehensive local hemostatic approach, contributed effectively to bleeding control
Bhavyaa, 2021 ^(20)^	India	Case report	A 7-year-old girl with GT	Dental extraction	A TXA tablet (500mg) was powdered, mixed with saline to form a paste, and placed into the socket. TXA mouthwash (500mg in 10mL water, 5%) was used six times daily for two days preoperatively	TXA paste applied to a soft plate effectively controlled bleeding in a patient with GT, highlighting its potential role in managing bleeding disorders
Souza et al., 2022 ^(17)^	Brazil	Retrospective study	Patients with liver disease (n=47)	Dental extraction and oral biopsy	TXA paste prepared from a crushed TXA tablet (250mg) mixed with saline was used to soak the RS filling the alveolar socket. For postoperative bleeding control, TXA mouthwash (one tablet in 100mL cold saline) was used four times daily for seven days	Patients with liver disease, thrombocytopenia, and a history of bleeding are at increased risk of hemorrhage. TXA, in combination with other local hemostatic measures, controlled bleeding and reduced the need for hospitalization

* Bleeding control was achieved using TXA in combination with additional local hemostatic measures.

DOAC: direct oral anticoagulant; GT: Glanzmann thrombasthenia; RS: resorbable sponge; TXA: tranexamic acid; VKA: vitamin K antagonist.

### Tranexamic acid references

Six studies used 250-mg TXA tablets,^(4,10,14–17)^ whereas three studies used 500-mg TXA tablets.^(18–20)^ Only one study applied TXA powder without specifying the exact concentration.^(21)^ These details are summarized in table 1.

### Tranexamic acid administration methods

Most studies reported the use of TXA as crushed tablets mixed with saline and applied as a paste directly to the surgical site, often in combination with gelatin or collagen hemostatic sponges.^(4,15-18,21)^ One study used TXA suspended in water and applied with wet cotton wool, a technique adapted for patients with hemophilia.^(19)^ In several studies, TXA paste mixed with saline was applied directly into the dental alveolus without the use of hemostatic sponges.^(10,14,20)^ These application methods are summarized in table 1.

For postoperative bleeding control, various TXA mouthwash formulations were described. Three studies recommended a TXA mouthwash prepared by dissolving one tablet in 100 mL of cold saline, used four times daily for seven postoperative days.^(15–17)^ Another protocol involved a mouthwash containing 500 mg of TXA used for two minutes, four times daily for four days.^(18)^ A different regimen recommended holding the mouthwash in the mouth for three minutes every six hours for seven days.^(14)^

### Population characteristics

Across the included studies, all participants had an underlying coagulation disorder. These conditions were medication-related, resulting from the use of antithrombotic agents, hereditary in origin, or associated with systemic diseases such as liver disease. Most studies involved individuals receiving antithrombotic therapy,^(15,21)^ including antiplatelet agents,^(10)^ vitamin K antagonists,^(4,14,18)^ or direct oral anticoagulants^(16)^ (Table 1). Two of the remaining studies focused on patients with hereditary bleeding disorders, namely hemophilia^(19)^ and Glanzmann thrombasthenia,^(20)^ whereas one study evaluated bleeding risk in patients with liver disease^(17)^ (Table 1).

### Types of dental procedures and their outcomes

Among the included studies, eight focused exclusively on bleeding risk associated with dental extractions,^(4,10,15,16,18-21)^ whereas one study also evaluated periodontal therapy^(14)^ and another included oral biopsy.^(17)^ These procedures are summarized in table 1.

All studies addressed bleeding management in individuals at high risk of hemorrhage undergoing dental surgical procedures. In every case, adjunctive local hemostatic measures, including topical application of TXA tablets, were effective in controlling bleeding (Table 1). Bernardoni-Socorro et al.^(14)^ reported successful bleeding control in anticoagulated patients using local measures combined with TXA. Coetzee^(19)^ identified crushed TXA tablets as a cost-effective option in resource-limited settings. Buhatem et al*.,*^(10)^ Rocha et al.,^(4,15,16)^ and Lu et al.^(21)^ demonstrated that TXA, often combined with other hemostatic measures, enabled safe dental treatment without interruption of anticoagulant or antiplatelet therapy. Bhavyaa et al.^(20)^ described the use of a TXA-soaked splint for bleeding control in a patient with Glanzmann thrombasthenia. Souza et al.^(17)^ showed that TXA effectively reduced bleeding complications, hospitalizations, and transfusion requirements in patients with liver disease (Table 1).

## DISCUSSION

In recent years, the effectiveness of hemostatic measures for controlling intraoperative and postoperative bleeding in anticoagulated patients has received increasing attention, with the aim of avoiding interruption of anticoagulant therapy during dental surgical procedures.^(22–24)^ The literature indicates that application of TXA in the form of crushed tablets, combined with fibrin sponges and mechanical gauze compression, effectively controls postoperative bleeding.^(25)^ Although evidence on this approach remains limited, this review represents the first synthesis focused specifically on the use of locally applied TXA powder, prepared from crushed tablets, for control of hemorrhagic events during dental surgery.

A systematic review of 129 randomized controlled trials demonstrated that TXA reduced the likelihood of blood transfusion by approximately one third^(.26)^ However, concern persists regarding potential thromboembolic risk, which may discourage some clinicians from adopting systemic administration in routine practice.^(26,27)^ In this context, topical application of TXA represents an effective alternative, with the advantage of inhibiting fibrin clot dissolution at the surgical site while limiting systemic exposure and interference with physiological coagulation processes.^(26,27)^ Findings from another review evaluating topical TXA reported significant reductions in bleeding and transfusion requirements among surgical patients. That analysis included 28 trials across multiple surgical disciplines, including orthopedics, cardiology, otolaryngology, and dentistry.^(26,27)^ Local application of TXA achieves hemostatic control while reducing risks associated with systemic administration, such as allergic reactions and renal overload, which occur more frequently with oral or intravenous routes.^(2,20,27)^

During the intraoperative period, TXA tablets have been explored as a hemostatic measure in minor oral surgical procedures. Tablets are crushed into powder and applied directly to the dental alveolus, increasing local drug concentration and enhancing bleeding control at the surgical site.^(10,14-17,20,28)^

Local administration of TXA in powder form may also represent a cost-effective alternative.^(29)^ Tablet formulations are substantially less expensive than intravenous preparations and are widely available, supporting their use in minor dental surgery.^(29)^ By reducing the need for blood transfusion and hospital-based interventions, TXA further contributes to lowering costs associated with hemorrhagic complications.^(27–29)^

Several pharmaceutical mouthwash formulations containing TXA have been proposed for control of postoperative bleeding following dental surgery. A systematic review including 430 anticoagulated individuals reported that local use of TXA significantly reduced postoperative bleeding risk, highlighting its role as an effective hemostatic measure in populations with coagulation disorders.^(30)^ When a commercially available 4.8% TXA mouthwash is not accessible in dental practice, an alternative approach involves preparation of a mouthwash using 250-mg or 500-mg TXA tablets dispersed in water.^(31–34)^ These formulations can be prepared by crushing tablets and dissolving them in purified water. To improve palatability, flavoring agents or aspartame may be added.^(2,35)^ Laboratory studies have demonstrated that such solutions remain stable and effective for up to 31 days when stored in bottles at temperatures of 23°C or 5°C.^(2)^

The reviewed literature indicates that plasma concentrations of TXA following topical application are less than one tenth of those achieved after intravenous administration, a finding considered advantageous because of reduced systemic absorption.^(26,27)^ In addition, comparison of a 5% TXA mouthwash with oral administration of a 1-g TXA tablet showed no detectable drug levels in saliva after oral dosing^(2)^. In contrast, rinsing with 10 mL of a 5% aqueous solution resulted in salivary concentrations sufficient to inhibit fibrinolysis for several hours.^(2,8)^

Within the limitations of this scoping review, topical TXA powder prepared from crushed tablets was most frequently applied in combination with other local hemostatic methods, including resorbable sponges, Gelfoam, sutures, and mechanical compression. Such combined strategies limit the ability to isolate and evaluate the independent effect of TXA. Nevertheless, the available evidence supports the role of TXA as a valuable adjunct for dental bleeding management and as an important component of intraoperative hemostatic protocols.

## CONCLUSION

The available literature indicates that topical application of tranexamic acid, as part of adjunctive local hemostatic measures, yields promising outcomes in controlling bleeding associated with minor oral surgery in individuals with coagulation disorders. However, further well-designed studies are required to evaluate the isolated efficacy of locally applied tranexamic acid powder in the management of bleeding complications following dental surgery.

## Data Availability

The underlying content is contained within the manuscript.
