# Supplementary material for: Topical tranexamic acid powder for bleeding control in dentistry: a scoping review
Source: Einstein (Sao Paulo). 2026 Mar 13;24:eRW1859. doi: 10.31744/einstein_journal/2026RW1859 (PMC13128248; doi:10.31744/einstein_journal/2026RW1859)
Supplement: Supplementary Material [file 2317-6385-eins-24-eRW1859-suppl01.pdf]

SUPPLEMENTARY MATERIAL

Topical tranexamic acid powder for bleeding control in dentistry: a scoping review

Beatriz Rezende Bergo, Letícia Mendes Nunes, Camila Vassallo de Andrade, Paula Cristina Costa, Francisca Daniele Moreira Jardilino, Amanda Leal Rocha

DOI: 10.31744/einstein_journal/2026RW1859

Table 1S. Comprehensive search strategy

Database	Descriptors	Search query
MEDLINE/PubMed	MeSH	("tranexamic acid powder" OR "crushed tranexamic pills") AND (oral surgery) AND (oral bleeding) AND ("dental extraction" OR "oral surgery")
Web of Science	MeSH	("tranexamic acid powder" OR "crushed tranexamic pills") AND (oral surgery) AND (oral bleeding) AND ("dental extraction" OR "oral surgery")
BVSalud	MeSH	("tranexamic acid powder" OR "crushed tranexamic pills") AND (oral surgery) AND (oral bleeding) AND ("dental extraction" OR "oral surgery")
Embase	DeCS	(('tranexamic acid'/exp OR 'tranexamic acid') AND 'tablet' AND (oral surgery) OR 'tooth extraction') AND 'oral bleeding)).
Scopus	DeCS/MeSH	("tranexamic acid") AND ("tablet" OR powder") AND ("oral surgery" OR "tooth extraction") AND ("oral bleeding")
Periódicos CAPES	DeCS	(tranexamic acid) AND ("oral surgery" OR "tooth extraction") AND (oral bleeding)

Table 2S. Critical appraisal of studies included in the review

Author, year	Study design	Assessment tool	Risk of bias
Bernardoni-Socorro et al., 1998[14]	Clinical trial	JBI Critical Appraisal Checklist	High
Blinder et al., 1999[18]	Clinical trial	JBI Critical Appraisal Checklist	High
Coetzee, 2007[19]	Letter to the editor	-	-
Buhatem Medeiros et al., 2017[10]	Case-control study	NOS	Low
Rocha et al., 2017[15]	Retrospective study	NOS	Low
Lu et al., 2018[21]	Retrospective study	NOS	Low
Rocha et al., 2019[4]	Prospective study	NOS	Low
Rocha et al., 2020[16]	Prospective study	NOS	Low
Bhavyaa, 2021[20]	Case report	JBI Critical Appraisal Checklist	Moderate
Souza et al., 2022[17]	Retrospective study	NOS	Low

NOS: Newcastle-Ottawa Scale; JBI: Joanna Briggs Institute.
